# The Role of Mesenchymal Stem Cells in the Induction of Cancer-Stem Cell Phenotype

**DOI:** 10.3389/fonc.2022.817971

**Published:** 2022-02-17

**Authors:** Yuanming Jing, Wenqing Liang, Lin Zhang, Junjun Tang, Zongliang Huang

**Affiliations:** ^1^ Department of Gastrointestinal Surgery, Shaoxing People’s Hospital (Shaoxing Hospital, Zhejiang University School of Medicine), Shaoxing, China; ^2^ Department of Orthopaedics, Zhoushan Hospital of Traditional Chinese Medicine Affiliated to Zhejiang Chinese Medical University, Zhoushan, China; ^3^ Department of Pharmacy, Shaoxing People’s Hospital, Shaoxing Hospital, Zhejiang University School of Medicine, Shaoxing, China; ^4^ Department of Radiology, Tongji Hospital, School of Medicine, Tongji University, Shanghai, China

**Keywords:** mesenchymal stem cells, cancer stem cells, cytokines, exosomes, tumor microenvironment

## Abstract

Cancer stem cells (CSCs) modify and form their microenvironment by recruiting and activating specific cell types such as mesenchymal stem cells (MSCs). Tumor-infiltrating MSCs help to establish a suitable tumor microenvironment for the restoration of CSCs and tumor progression. In addition, crosstalk between cancer cells and MSCs in the microenvironment induces a CSC phenotype in cancer cells. Many mechanisms are involved in crosstalk between CSCs/cancer cells and MSCs including cell-cell interaction, secretion of exosomes, and paracrine secretion of several molecules including inflammatory mediators, cytokines, and growth factors. Since this crosstalk may contribute to drug resistance, metastasis, and tumor growth, it is suggested that blockade of the crosstalk between MSCs and CSCs/cancer cells can provide a new avenue to improving the cancer therapeutic tools. In this review, we will discuss the role of MSCs in the induction of cancer stem cell phenotype and the restoration of CSCs. We also discuss targeting the crosstalk between MSCs and CSCs/cancer cells as a therapeutic strategy.

## 1 Introduction

Cancer stem cells (CSCs), which have been shown to play a vital role in tumor origin, are considered to be responsible for tumor progression, drug resistance, and metastasis (1). CSCs can form their microenvironment by recruiting and activating specific cell types such as mesenchymal stem cells (MSCs). Then, MSCs can modify the stroma and establish a unique tissue microenvironment that is suitable for the restoration of CSCs and tumor progression (2). In addition, crosstalk between cancer cells and MSCs in the microenvironment can induce a CSC phenotype in cancer cells. Many mechanisms are involved in crosstalk between tumor cells and MSCs including cell-cell interaction, secretion of exosomes, and paracrine secretion of several molecules including inflammatory mediators, cytokines, and growth factors (3). Since crosstalk between tumor cells and MSCs may contribute to drug resistance, metastasis, and tumor growth, it is suggested that blockade of the crosstalk between MSCs and tumor cells can provide a new avenue to improving the cancer therapeutic tools.

Many studies show the crosstalk between tumor cells and MSCs. For instance, transforming growth factor (TGF)-β-stimulated MSCs can induce a metastatic phenotype by upregulating Jagged-1, a major ligand of Notch signaling, in tumor cells (4). Indeed, activation of the Notch signaling pathway induces epithelial-mesenchymal transition (EMT) and promotes a cancer stem cell phenotype. This phenomenon is supported by other studies that show the relationship between the EMT process and CSCs (5). In another study, in hepatocellular carcinoma, treating MSCs with tumor necrosis factor-α (TNF-α) and interferon γ (IFNγ) causes an increase in production of TGFβ by MSCs which in turn could promote tumor metastasis by inducing EMT in cancer cells (6). Luo, et al. have reported that the increased metastatic phenotype of prostate cancer (PCa) cells could be due to an increase in the PCa stem cell population. They showed that increase in the stem cell population is mediated by MSCs through alteration of the CCL5–AR signaling pathway (7). Indeed, the upregulation of CCL5 in bone marrow mesenchymal stem cells (BM-MSCs) and PCa cells, after MSCs infiltrated into PCa microenvironment, lead to downregulation of the androgen receptor (AR) signaling pathway (7). Increasing in the PCa stem cell populations led to the upregulation of CXCR4, ZEB-1, matrix metallopeptidase 9 (MMP-9), and CD133 that these molecules promote the metastatic phenotype of PCa cells (7). Recently, Hossain et al. reported that in glioblastomas, tumor-associated mesenchymal stromal cells promote the proliferative and tumorigenic phenotype of glioma cancer stem cells (gCSCs) through the IL-6/STAT3 signaling pathway (8). The aggressiveness of gCSCs is enhanced in co-culture with MSCs, and these observations were supported by reduced survival in orthotopic xenograft mouse models, increased cell counts *in vitro*, enhanced angiogenesis, and tumor size *in vivo* (9). Gene expression analysis of cancer-associated (CA)-MSCs revealed that they can alter synthesis levels of bone morphogenetic protein (BMP) signaling pathway proteins. BMP2 can significantly increase the number of CSCs in primary ovarian tumor cells and ovarian cancer cell lines (10). *In vivo* and *in vitro* suppression of the BMP signaling pathway with Noggin suppress the capability of CA-MSCs to support tumor growth and tumor stemness (10). Thus, MSCs can enhance tumorigenesis, at least in part, through the promotion of the BMP signaling pathway (10). On the other hand, Vulcano et al. reported that Wharton’s jelly of umbilical cord (WJMSC) exert, both *in vivo* and *in vitro*, conflicting impacts on lung cancer stem cells derived from various lung cancer subtypes (11).

In this review, we will discuss the role of MSCs in the induction of cancer stem cell phenotype and the restoration of CSCs. We also discuss targeting the crosstalk between MSCs and CSCs/cancer cells as a therapeutic strategy.

## 2 MSCs Mediated Mechanisms of Increasing CSC Population

Various mechanisms are involved in inducing the stem cell phenotype in tumor cells and restoring of CSCs including cell fusion, direct transformation of MSCs into CSCs, crosstalk of MSCs with CSCs/tumor cells mediated by secretory factors, exosomes, etc. We will go into the details of the mentioned mechanisms in the following.

### 2.1 MSCs Secreted Factors/Cancer Cell Contact and CSCs

Plenty of studies has been performed to indicate how cellular components of the cancer microenvironment participate in cancer development. CSCs by recruiting and activating specific cell types establish their microenvironment. MSCs are one of the main cellular components which release various cytokines that have both autocrine and paracrine functions in the cancer milieu (2).

#### 2.1.1 MSCs Secreted Factors/Cancer Cell Contact and Induction of CSC Phenotype

**Figure 1:**
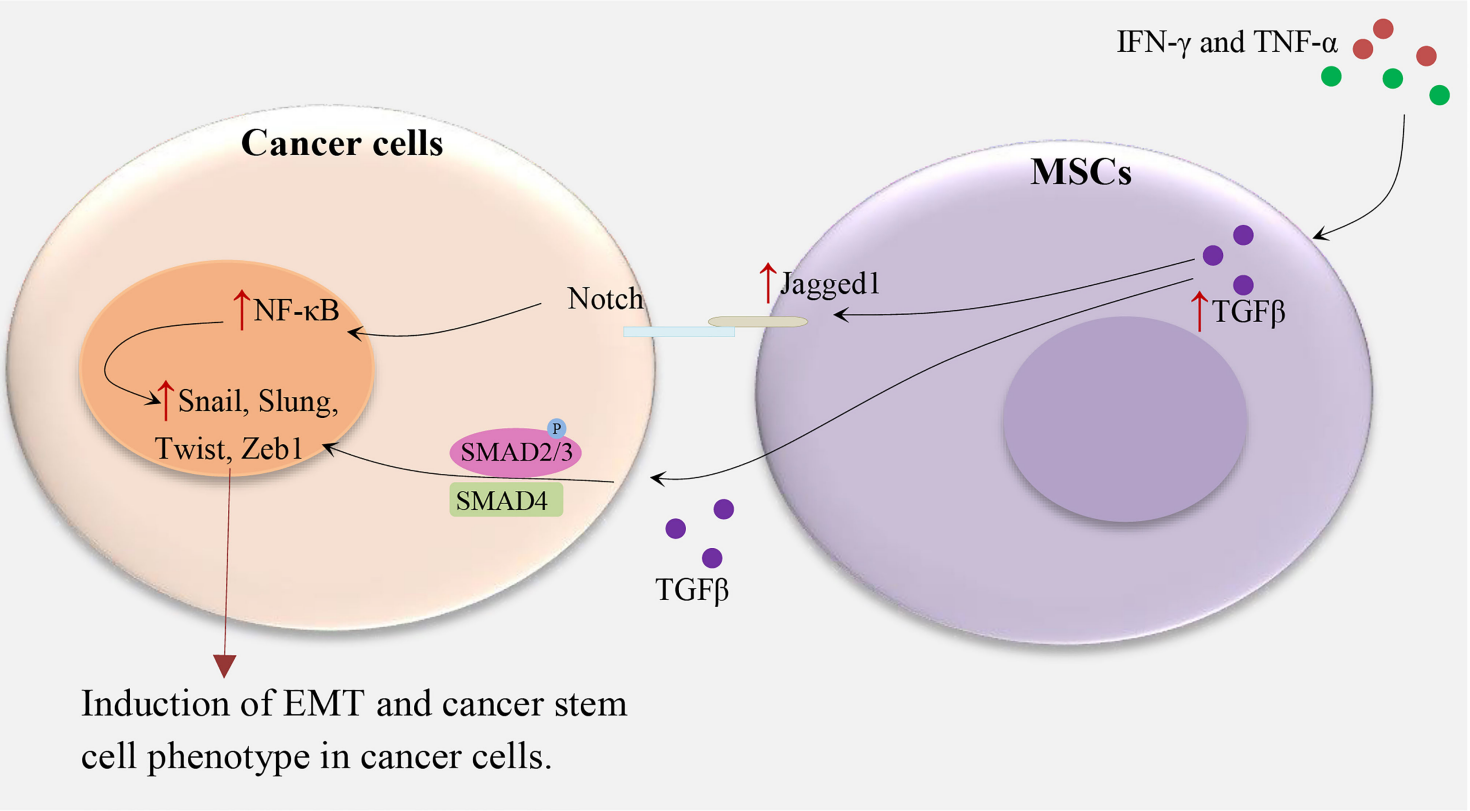
MSCs and induction of CSC phenotype in cancer cells. IFN-γ and TNF-α lead to TGFβ overexpression in MSCs, subsequently, TGFβ upregulates Notch signaling and TGF-β/Smad signaling pathways in cancer cells and induce the cancer stem cell phenotype by upregulating Zeb1, Twist, Slug, Snail, and others which are related to EMT (4, 6, 12, 13).

MSCs promote EMT by the secretion of cytokines and growth factors such as TGFβ (**Figure 1**). These factors stimulate transcriptional regulators, such as Zeb1, Twist, Slug, Snail, and others which are related to EMT (12, 13). For instance, in hepatocellular carcinoma, treatment of MSCs with IFNγ and TNFα leads to a high expression level of TGFβ which in turn induces EMT-related properties in tumor cells (6). In another study, TGFβ-induced MSCs in pancreatic cancer increase the metastatic potential by upregulating Jagged-1, a major ligand of Notch signaling in cancer cells (4). In turn, stimulation of the Notch signaling pathway promotes EMT and induces a CSC phenotype. Some other studies supported the role of EMT in the induction of CSC phenotype (5). An alternative mechanism of MSC-induced CSC phenotype has been indicated in gastric cancer. MSCs are recruited by gastric mucosal cells infected with *Helicobacter* which then transform into gastric cells expressing epithelial biomarkers including TFF2 and KRT1-19. Therefore, chronic inflammation induces the CSC properties of gastric cancer by inducing the EMT and metastatic phenotype (14).

**Figure 2:**
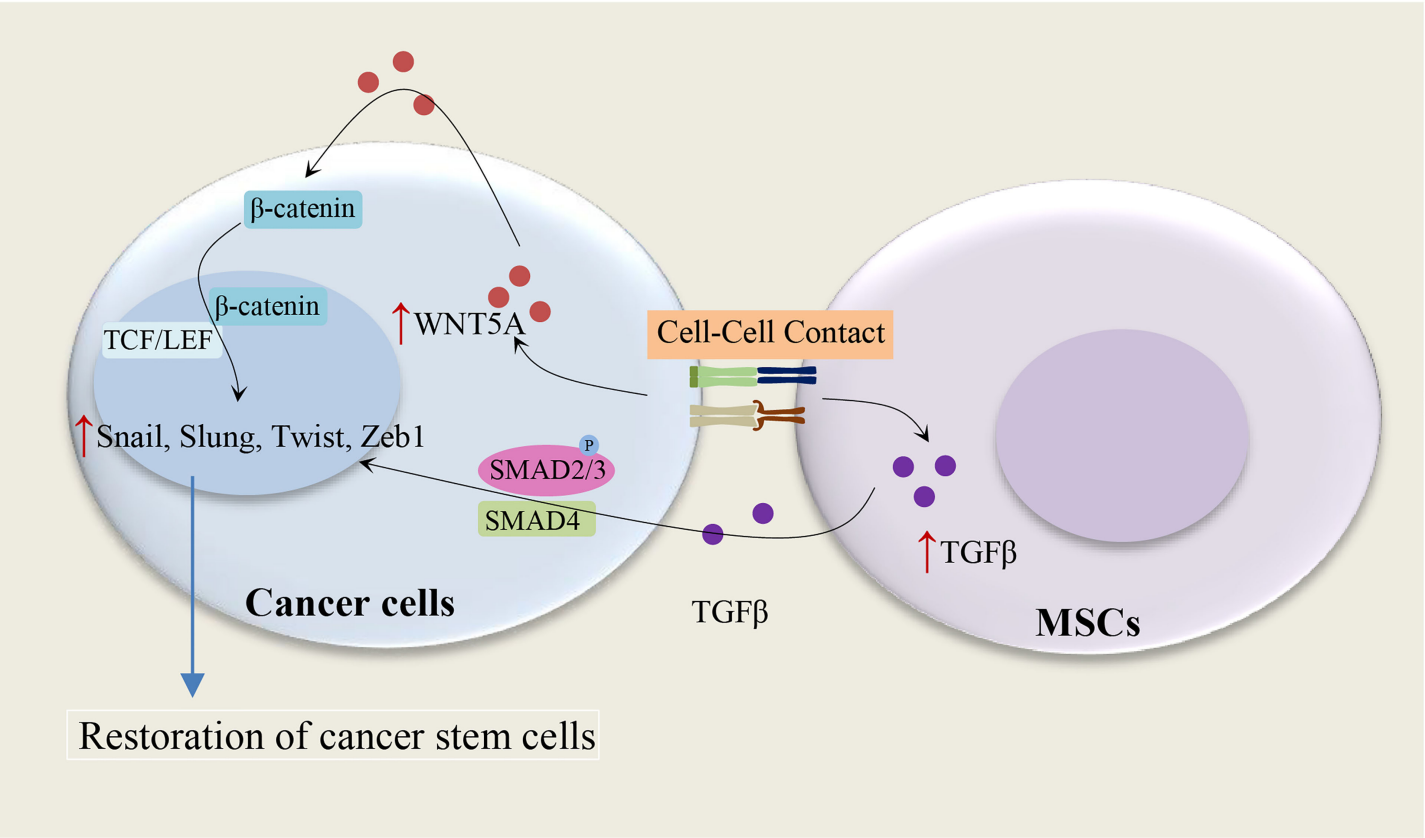
Cell-cell contact and restoration of CSC populations. Cell contact between MSCs and cancer cells leads to an increase of TGF-β1 in MSCs and WNT5A in cancer cells, subsequently, the paracrine effect of TGF-β1 and autocrine effects of WNT5A on cancer cells result in the restoration of CSC populations. WNT5A and TGF-β enhance the expression of the Snail-family transcription factors, including *Slug*, *Snail*, *Twist1*, and *Twist2* (15–17).

The MSC-induced paracrine effect of TGF-β1 and autocrine effects of WNT5A on the restoration of CD133^+^ CSC populations show the importance of the tumor microenvironment for the maintenance of CSCs (15), as illustrated in **Figure 2**. The autocrine effects of WNT5A in gastric carcinoma cells can contribute to the activation of the WNT-β-catenin signaling pathway (15). It has been shown that both TGF-β and WNT5A play a crucial role in the stimulation of EMT in tumor cells: WNT5A stable melanoma cells transfectants indicate a spindle shape accompanied by increased vimentin expression and decreased E-cadherin expression (16), and TGF-beta mediated EMT is regulated by the SNAIL1-SMAD3/4 transcriptional complex, which acts as a suppressor of E-cadherin expression (17). Indeed, WNT5A and TGF-β significantly enhance the expression of the Snail-family transcription factors, including *Slug*, *Snail*, *Twist1*, and *Twist2* (15). These studies suggest a direct relationship between the EMT and the gain of CSC phenotype (18).

#### 2.1.2 MSCs Secreted IL-1, IL-6, PEG-2, and Induction of CSCs

**Figure 3:**
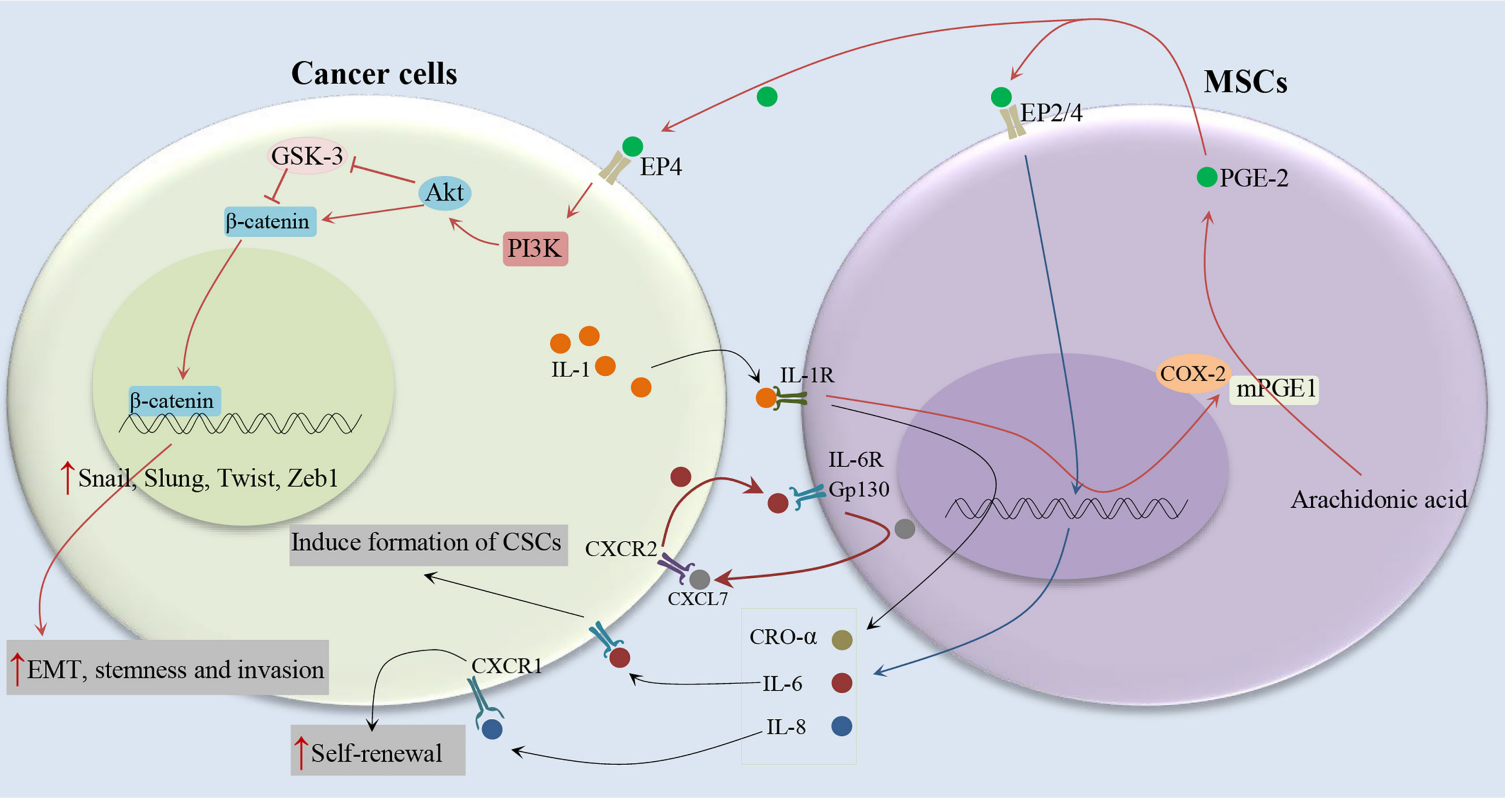
Cytokine networks between MSCs and cancer cells and induction of CSC phenotype. Cancer cells stimulate the COX-2/mPGES-1/PGE 2 axis in MSCs by releasing IL-1. MSCs release PGE-2 in response to IL-1, PGE-2 in an autocrine fashion promote the expression of IL-8, IL-6, CXCL1, RANTES, and GRO-α, which together stimulate the formation of CSCs (19–21).

It has been shown that IL-1 released by head and neck squamous cell carcinoma (HNSCC) cells stimulates prostaglandin-E2 (PGE-2) from fibroblasts (19), as illustrated in **Figure 3**. It has been also reported that cancer cells are able to provoke a strong stimulation of the cyclooxygenase-2 (COX-2)/microsomal Prostaglandin-E synthase-1 (mPGES-1)/PGE 2 axis in MSCs recruited to the cancer-associated stroma by releasing IL-1 (20). The tumor-promoting effects of COX-2 are mostly related to its role in inducing PGE-2, which has pleiotropic effects on invasiveness, angiogenesis, motility, survival, and cell proliferation (21). It is found that IL-1 plays a critical role in the cancer cell-induced COX-2/mPGES1/PGDH/PGE 2 response in MSCs that is necessary for tumor development (20). In colorectal cancer, MSCs release PGE-2 in response to IL-1 secreted by tumor cells, PGE-2 in an autocrine fashion promotes the expression of IL-8, IL-6, CXCL1, RANTES, and GRO-α, which together stimulate the formation of CSCs (20). PGE-2, which can trigger the EMT phenotype, promote both the frequency of cancer initiation and the number of CSCs (20). Li et al. (20) showed the partial EMT phenotype induced by PGE 2 suffices to increase CSCs by inducing a stem cell-like phenotype in cancer cells by suppressing cell-cell junctions without stimulating mesenchymal traits (20). Previous studies have shown the role of prostaglandin E2 in increasing the number of CD44^+^ cancer cells (22, 23). Observations show that other MSC-derived cytokines, compared to PEG-2, have marginal effects on the increasing tumor-initiating cell frequency (20). The PGE-2 and cytokines act in a paracrine fashion on the tumor cells to stimulate the β-catenin signaling pathway and formation of CSCs. Therefore, MSCs and derived cell types construct a CSC niche and promote cancer progression *via* the secretion of PGE-2 and other cytokines (20). IL-1 blocking therapies are used in the clinic to control inflammatory and infectious diseases and have a remarkable safety record (24). IL-1 blocking may provide a promising alternative to COX-2 inhibitors in cancer therapy (20).

It has been indicated the association between serum IL-6 levels and poor clinical outcomes of breast cancer patients (25, 26). IL-6 acts as a direct regulator of CSC self-renewal, a process triggered by the IL-6 receptor/GP130 complex through the activation of STAT3 (27). On the other hand, It has been reported that MSCs can secrete IL-6 (28, 29), and induce tumor growth through the paracrine function (30). Liu et al. (31) indicated that the communication between CSCs and MSCs is facilitated by a positive feedback cytokine loop in which CXCL7 and IL6 play essential roles (31), as illustrated in **Figure 3**. This loop needs the simultaneous presence of these cells but does not require direct interactions between cell surfaces as indicated by trans-well and conditioned medium experiments (31). IL6 secreted by tumor cells interacts with gp130 and interleukin 6 receptor (IL-6R) on ALDEFLUOR-positive mesenchymal cells (MCs) and can promote the homing of MSCs to the tumor sites, as well as stimulate CXCL7 expression by these cells. Then, MSC-derived CXCL7 interacts with tumor cells through the CXCR2 receptor (32), where it stimulates the production of some cytokines, such as IL-8 and IL-6 (32). IL-6 secreted by tumor cells interacts with MSCs and further enhances their CXCL7 expression, therefore generate a positive feedback loop. This cytokine loops between BCSCs and MSCs promote the self-renewal of BCSCs (31). Moreover, it has been shown that CXCL7 transfection increases the invasive ability of cancer cells (33), consistent with the previous findings that showed an increase in metastatic and invasive properties of CSCs (34). Furthermore, Sethi et al. indicated that IL-6–mediated Jagged1/Notch signaling induces breast cancer bone metastasis (35). These findings introduce IL-6 and its receptor as attractive therapeutic targets. It has been shown that CXCR1, a receptor for IL-8, and IL-8 can induce their self-renewal (36). In addition, it has been reported that the interaction of IL-8 with the CXCR1 (highly expressed on breast cancer stem cells), on CSCs increases their invasive and self-renewal properties (36, 37). Blocking the CXCR1 in mouse xenograft models significantly decreases the number of BCSCs, leading to reduced metastasis and tumorigenicity.

#### 2.1.3 Crosstalk Between CSCs and MSCs Mediated by Exosomes

The crosstalk between stromal cells and CSCs are facilitated by cell-cell interaction and paracrine factors (2). In addition, cellular crosstalk has also been reported to be facilitated by the secretion of extracellular vesicles (EVs) that can transfer nucleic acids, lipids, and proteins and are able to induce epigenetic changes in target cells (38–40). This EV-mediated crosstalk is associated with chemoresistance, tumor development, and the capacity of evading from immune surveillance (41–44). Numerous studies have shown that cancer EVs are not limited only to the cancer microenvironment but there are also in body fluids such as blood circulation, emphasizing the idea that these vesicles can also influence the cells in other tissues (45–48). Peinado et al. indicated that exosomes secreted by melanoma can “educated” bone marrow progenitor cells to promote metastatic phenotype (49). Stem cells likely can alter the expression of genes in neighboring cells through exosomes containing microRNAs (miRNAs) (50).

It has been shown that stimulated MSCs with CSC-derived EVs considerably increase the migratory ability in response to cancer chemo-attractive stimuli. Indeed, CSC-EVs increase the expression of migration process-related genes. CXCR4, which is increased after CSC-EV stimulation, enhances the migratory capacity of MSCs toward the tumor site through an SDF-1 concentration gradient secreted by cancer cells (51–53). Stimulated MSCs also show an increased *CXCR7* expression, an SDF-1 receptor associated with paracrine actions of MSCs (51, 54, 55). The ability of MSCs to modify extracellular matrix within the cancer microenvironment has been confirmed by increased expression of *MMP1*, *2*, and *3* after stimulation with CSC-EVs. MMPs are proteolytic enzymes that are associated with metastasis processes, invasion, tumor growth, and angiogenesis (56). Tumor matrix remodeling activity of stimulated MSCs has also been confirmed by the increase of *COL4A3* gene expression (51). This gene is involved in regulating cell adhesion, migration, and metastasis in various cancer types (57–59). EVs secreted from CSC rather than total cancer cell population show a central role in inducing pro-tumorigenic phenotype in MSCs (51).

Upon stimulation with CSC-EVs, the secretory profile of MSCs is changed and expression of IL-8, myeloperoxidase (MPO), and osteopontin (OPN) are increased (51). MPO has been shown to be involved in oxidative stress response and the anti-apoptotic process by converting nitric oxide (NO) into NO(+) that induces an S-nitrosylation of caspase-3, inhibiting its activity (60, 61). In clear cell renal cell carcinoma, OPN by stimulating of NF-κB and protecting cells from apoptosis induces tumor development (62). OPN also shows an autocrine function on MSCs by stimulating extracellular signal-regulated kinase (ERK) and focal adhesion kinase (FAK) signaling pathways *via* β1-integrin activation. This leads to the increase of MSCs motility and subsequently promotes the migration of MSC (63).

The anti-tumor activity of MSCs may depend on the type or even stage of cancer. Whereas naive MSCs may demonstrate an anti-cancer activity (64–66), indeed pre-conditioning of MSCs by cancer EVs may change their phenotype and function. Therefore, it is recommended that the secretion of cancer EVs be pharmacologically inhibited for preventing their unwanted effects before the administration of MSCs in cell-based cancer therapy approaches (67, 68). The phenotypic changes in MSCs mediated by CSC-derived EVs are maintained even after removal of stimulation, suggesting a persistent change in MSC phenotype (51).

#### 2.1.4 CAFs Originated From MSCs and Cancer Stemness

Although the majority of tumor tissue cancer-associated fibroblasts (CAFs) may originate from resident stromal fibroblasts, but now many studies show that a significant percentage of CAFs in cancers are originated from BM-MSCs. For instance, MSCs in mouse models of gastric cancer, breast cancer, and PDAC, BM-MSCs are recruited to the cancer site where they differentiate into CAFs (69–71). It has been reported that in a gastric cancer model, about 20% of CAFs in tumor sites are derived from BM-MSCs which have been recruited into the tumor microenvironment in a TGF-β and CXCL-12 dependent manner (72).

The pro-stemness abilities of CAFs are one of the main mechanisms that increase oncogenesis. A specific subpopulation of CAFs has been identified that are proficient in secreting pro-stemness paracrine factors (73–79), thereby supporting the stemness properties and the self-renewal of tumor resident CSCs or increasing the transformation of tumor cells into CSCs. When exposed to cytotoxic stress such as chemotherapy, CAFs are further stimulated to produce pro-stemness cytokines or obtain a senescence-like secretory phenotype and secrete large amounts of pro-stemness chemokines that increase cancer stemness and aggressiveness following cancer treatment (80, 81). Therefore, blockade of the crosstalk of CSCs/cancer cells with pro-stemness CAFs and MSCs may introduce a new tool to improving the clinical outcome of solid tumors.

### 2.2 Cell Fusion and CSC-Like Phenotype

It has been shown that the biological phenomenon of cell fusion plays an important role in several pathological and physiological processes (82, 83). Cell fusion of stromal cells with tumor cells has been confirmed in human tumors and animal models (84–86). Studies have demonstrated that hybrid cells resulting from a spontaneous fusion between cancer cells and MSCs indicate metastatic, tumorigenic, and stem cell-like properties (87–89) and this phenomenon leads to nuclear reprogramming (90). In addition, the theory of cell fusion may clarify the origin of CSCs and the mechanism for cancer metastasis and carcinogenesis. He et al. (91) reported that cell fusion of gastric epithelial cells with MSCs promotes proliferation, migration, and invasion capabilities compared with the parental cells (91). The fusion of MSCs with HepG2 also promotes the malignant properties of *in vivo* metastasis models (92). In contrast, some findings demonstrate that the fusion of MSCs with esophageal carcinoma cells suppresses tumorigenicity (93). It has been also reported that the fusion of tumor cells with normal fibroblasts inhibits the tumorigenicity through cell cycle arrest effects (94). Similar results have been shown in cell fusion studies of stem cells and tumor cells (93, 95). The cell fusion hypothesis of the CSCs suggests that the fusion process may lead to the EMT of tumor cells and simultaneously promote the generation of CSCs (96). Zhang et al. demonstrated that fusion between MSCs and lung tumor cells may directly activate EMT of the hybrids, which promotes the invasion and migration properties (97). Meanwhile, the overexpression of stem cell surface markers (CD44 and CD133), and stem cell transcription factors (Kif4, Oct4, Sox2, Nanog, and Bmi1) in hybrids, show that the hybrids may obtain CSC properties after cell fusion (97). Although the hybrids show stem cell-like properties, further studies are required to determine whether the hybrids are the origin of CSCs.

### 2.3 Transformation of MSCs Into CSCs

The origin of cancer-initiating cells or CSCs has not been clearly determined. CSCs may be derived either from dedifferentiated mature cells or from transformed somatic stem cells (98–104). It has been shown that specific methylation of tumor suppressor genes, HIC1 and RasF1A, in MSCs can lead to the transformation of MSCs into CSCs. When MSCs transform into CSCs, they can increase drug resistance and allow tumor recurrence after treatment cessation (105). Indeed, abnormal DNA methylation of RassF1A and HIC1 is involved in the transformation of MSCs to cancer-like stem cells. Concurrent methylation of RassF1A and HIC1 has been reported in advanced ovarian cancer (106), and HIC1 demonstrates enhanced concordant hypermethylation with other genes in advanced myelodysplasia syndrome (107), suggesting that disruption of HIC1-associated networks may be critical for cancer initiation. Unlike RassF1A, which can be inactivated by either epigenetic or genetic mechanisms, suppression of the HIC1 gene is mainly caused by DNA methylation (108). Thus, specific methylation of HIC1 could predispose cells to tumor development. A subsequent epigenetic/genetic hit, such as RassF1A methylation, may then permit more efficient tumor progression. Concordant silencing of RassF1A and HIC1 may synergistically interrupt the p53 Pathway for apoptosis signaling and involve in the observed tumorigenic capability of MSCs. Teng et al. (105) showed that forced epigenetic silencing of RassF1A and HIC1 is adequate to induce malignant properties, including migration ability, enhanced colony formation, chemoresistance, loss of contact inhibition, and tumor formation in normal somatic stem cells (105). In addition, MSCs also reprogram toward CSCs, due to the aberrant changes of tumor microenvironments, which leads to the tumor development through the increased production of Oct4, Sox2, Nanog and the activation of Hedgehog, Wnt, Akt/mTOR, and NF-kB signaling pathways (31, 109–112). It has been reported that EWS-FLI-1 fusion protein modulates the expression of CSC signature proteins such as Oct4, Nanog, and Sox2 in MSCs that can reprogram these cells toward Ewing sarcoma CSCs (112). It has been also shown that MSCs cultured with cancer cell-soluble factors show a cancer stem cell-like state (113).

## 3 Cancer Treatment by Targeting CSCs, MSCs, and Their Crosstalk

MSCs help to establish a suitable tumor microenvironment for the restoration of CSCs and tumor progression, as well as crosstalk between cancer cells and MSCs in the microenvironment induces a CSC phenotype in cancer cells. Since this communication can contribute to drug resistance, metastasis, and tumor growth, it is suggested that blockade of the crosstalk between MSCs and CSCs/cancer cells can provide a new avenue to improving the cancer therapeutic tools. Here, we discussed various strategies in targeting the crosstalk between MSCs and CSCs/cancer cells (**Tables 1**, **2**).

**Table 1:** Targeting approaches of crosstalk between MSCs and CSCs.

Targeting Approach	Molecular/Cellular Target	Description	Reference
**Targeting the molecular crosstalk between cells**	CXCR3 antagonist (AMG487) with “nano-ghost (NG)”	Cytotoxic agent exposed MSCs secrete high levels of CXCL-10 that stimulate its receptor CXCR-3 on CSCs, triggering STAT-3 signaling pathway and supporting the survival of CSCs	(114)
	Blocking the IL-1	A promising alternative to COX-2 inhibitors in cancer therapy. IL-1 secreted by MSCs induce the CSC phenotype	(20, 24)
	Blocking IL-6 and its receptor	MSCs release the pro-stemness cytokine IL-6, the various STAT-3 inhibitors and/or anti-IL-6 antibodies exploited to blockade the CAF/MSC–CSC crosstalk	(36, 74, 115, 116)
	Blocking the CXCR1	Blocking the CXCR1 significantly decreased the number of CSCs	(117)
**Direct targeting of cells**	Targeting CAFs/MSCs	CAFs can account for more than 90% of the total cancer size. CAFs are often localized to the margin of the glands or the cancer cell nests and close to blood vessels, therefore, drug delivery to these cells is easy.	(118–120)
	Dual targeting of CAFs and MSCs	synergistic effect and maximize the anticancer efficacy in the treatment of desmoplastic cancers.	(121)
	Targeting pro-stemness CAFs/MSCs	Because of the dynamic, heterogeneous, and plastic properties of CSCs, targeting the CAFs/MSCs is more reasonable than the direct targeting of CSCs. Targeting the specific subpopulations of pro-stemness CAFs will provide more specific and safer therapies than the non-specific targeting of CAFs	(122, 123)
	Specifically targeting of CSCs by TRAIL-expressing MSCs	TNF-related apoptosis-inducing ligand (TRAIL)-expressing MSCs specifically target CSCs	(124, 125)
	Low-dose metronomic (LDM) chemotherapy	at least reduce chemotherapy-induced stimulation of MSCs and their production of pro-stemness chemokines such as CXCL-10	(81)
	Targeting the CSCs by using the exosomes	Exosomes derived from MSCs containing exogenous LNA-antimiR-142-3p to inhibit miR-142-3p	(126)

**Table 2:** Agents targeting CSC, MSC, and CSC-MSC crosstalk in clinical trials.

Drug/agent Name	Drug Target	Cancer Type	Phase	Current Status	NCT Number
**Surface antigens of CSC**					
Removab	EpCAM/CD3	Ovarian cancer	II	Completed	NCT00189345
Talacotuzumab	CD123	Acute myeloid leukemia	II/III	Completed	NCT02472145
Mylotarg	CD33	CD33+ R/R AML	IV	Completed	NCT03727750
**Immune checkpoints**					
Atezolizumab	PD-L1	NSCLC	III	Completed	NCT02008227
Ipilimumab	CTLA-4	NSCLC	II	Completed	NCT01820754
Varlilumab	CD27	Advanced refractory solid tumors	I/II	Completed	NCT02335918
**Hedgehog inhibitors**					
Vismodegib	Smoothened	Metastatic colorectal cancer	II	Completed	NCT00636610
Sonidegib	Smoothened	Basal cell carcinoma syndrome	II	Completed	NCT01350115
**Notch inhibitors**					
MK-0752	γ-Secretase	Advanced or metastatic sarcoma	I/II	Completed	NCT01154452
Demcizumab	DLL4	Metastatic pancreatic ductal adenocarcinoma	II	Completed	NCT02289898
**Wnt inhibitors**					
Ipafricept	Wnt receptor	Solid tumors	I	Completed	NCT01608867
PRI-724	β-Catenin/CBP	Acute myeloid leukemia	I/II	Completed	NCT01606579
**Other signaling pathways inhibitors**					
Galunisertib	TGF-β	Prostate cancer	II	Recruiting	NCT02452008
Ruxolitinib	JAK	Breast cancer	II	Completed	NCT01594216
**Niche inhibitors**					
Plerixafor	CXCR4	Multiple myeloma	I/II	Completed	NCT01010880
BL-8040	CXCR4	Metastatic pancreatic adenocarcinoma	II	Active, not recruiting	NCT02907099
**CSC-directed immunotherapy**					
CD19 CAR-T	CD19+ cells	B cell leukemia and lymphoma	I/II	Recruiting	NCT03398967
MESO-19 CAR-T		Metastatic pancreatic cancer	I	Terminated	NCT02465983
LeY-targeted CAR-T		Myeloid malignancies	I/II	Unknown	NCT02958384
BCMA CAR-T		Multiple myeloma	I/II	Recruiting	NCT03767751
**MSCs−based cancer therapy**					
MSC		Hematological malignancies	II	Terminated	NCT01045382
CELYVIR		Metastatic and refractory tumors	I/II	Completed	NCT01844661
MSC-TRAIL		Lung adenocarcinoma	I/II	Recruiting	NCT03298763
BM-MSCs-DNX2401		Glioma	I	Recruiting	NCT03896568

### 3.1 Chemotherapy-Educated MSCs and CSCs

Exposure of MSCs to cytotoxic agents resulted in a physiological response in these cells that eventually supports chemoresistance by enriching the CSC population. It has been reported that MSCs are recruited in large numbers to the tumor site in response to gemcitabine treatment. Gemcitabine-exposed MSCs, which are located near CSCs and support the CSC niche, significantly increase the secretion of CXCL10, which in turn induces CSCs proliferation as they overexpress CXCR3, the CXCL10 receptor. These events ultimately lead to increased tumor growth and drug resistance (114). It has been also shown that in a mouse xenograft model of PDAC, the number of BM-MSCs significantly increases following gemcitabine therapy in the tumor stroma (114). These gemcitabine-educated MSCs present a positive regulatory effect on cancer stem cells through the STAT-3: CXCL-10: CXCR-3 paracrine signaling axis. Similarly, MSCs secrete IL-8, IL-6, IL-7, IGF, and EGF, which promote chemoresistance following hyperthermia or paclitaxel therapy (127, 128). It has been also reported that cisplatin-exposed MSCs release specific polyunsaturated fatty acids which in turn increase the regrowth of cancers following treatment (129). In addition, exposure with cisplatin changes phosphorylation of several tyrosine kinases, including c-Jun, WNK-1, p53, and STAT3 in MSCs, and promotes MSC survival and secretion of IL-8 and IL-6. In turn, these events induce chemoresistance of cancer cells (130). However, the exact mechanisms by which MSCs promote chemoresistance have not been identified. Altogether, ample evidence emphasizes the central role of MSCs and CAFs in the expansion and maintenance of CSCs. Thus, targeting these cells may provide a new way to improve the clinical outcome of desmoplastic cancers.

### 3.2 Targeting the Crosstalk Between MSCs and CSCs

Due to the pro-tumorigenic activities of MSCs, a number of studies had been conducted to target MSCs as a therapeutic method in cancer (131). Because tumor-infiltrating MSCs can directly support cancer stem cells through several paracrine signaling pathways, including IL-7, IL-6, Jagged-1, PGE-2, CXCL-1, and CXCL-10 (4, 20, 114, 132), blockade of this paracrine communication between CSCs and MSCs may be potentially valuable in inhibiting tumor stemness in solid tumors. Indeed, a recent study has demonstrated the potential application of this approach (114). In a mouse model of PDAC, MSCs are located near CSCs, the CSC niche, following gemcitabine treatment. Gemcitabine-exposed MSCs release high levels of CXCL-10 that stimulate its receptor CXCR-3 on CSCs, triggering the STAT-3 signaling pathway and supporting the survival of CSCs (114). It has been reported that systemic injection of the CXCR3 antagonist (AMG487) with “nano-ghost (NG)”, MSC-derived membrane-based nanoparticles, leads to its accumulation in the CSC niche, thus decreasing the number of CSCs and enhancing the therapeutic efficacy of gemcitabine (114). The direct depletion of MSCs may be an alternative approach to preventing their communication with CSCs. However, the negative effects of the removal of MSCs on a person’s health remain an open question. Alternatively, it has been shown that low-dose metronomic (LDM) gemcitabine therapy regimen can decrease therapy-induced secretion of pro-stemness factors from CAFs in PDAC (81). Therefore, it is likely that LDM chemotherapy may also at least reduce chemotherapy-induced stimulation of MSCs and their production of pro-stemness chemokines such as CXCL-10. This possibility needs further investigation. On the other hand, since MSCs release the pro-stemness cytokine IL-6 (20), the various STAT-3 inhibitors and/or anti-IL-6 antibodies are exploited to blockade the CAF/MSC–CSC crosstalk (74, 115, 116, 133). It is anticipated that dual targeting of CAFs and MSCs may have a synergistic effect and maximize the anticancer efficacy in the treatment of desmoplastic cancers (121). Targeting approaches of crosstalk between MSCs and CSCs are summarized in **Tables 1**, **2**.

### 3.3 Targeting Pro-Stemness CAFs and MSCs

Unlike direct targeting of CSCs, which poses substantial challenges such as dynamic and plastic properties of CSCs, targeting the MSCs or CAFs along with the pro-stemness niches they create can have several advantages in cancer therapy. First and foremost, there is ample evidence to show that CSCs are very plastic and heterogeneous and the transformation between different CSC populations plays a key role in cancer development and treatment response (122). For example, breast cancer CSCs can transition between epithelial-like states and mesenchymal-like (134–136). CSCs can also be originated from differentiated tumor cells through transdifferentiation or cellular reprogramming (73), which can be facilitated by cytotoxic agents such as ionizing radiation and chemotherapy (81, 137). It has been shown that eradicating LGR-5^+^ CSCs suppresses tumor growth, whereas the regrowth of cancer occurs following the removal of the cell death inducers due to the regeneration of CSCs from differentiated cancer cells (137, 138). Unlike CSCs, CAFs are both phenotypically and genetically stable; thus, CAF-targeted treatments may result in a more stable anti-CSC effect compared with direct targeting of CSCs. Second, identification of specific subpopulations of pro-stemness CAFs will facilitate CAF-targeted therapy, and they not only render new therapeutic targets, such as GPR-77 (123) but also provide more specific and safer therapies than the non-specific targeting of CAFs (139). In desmoplastic cancers such as pancreatic cancer, CAFs are present in large numbers in the stroma, which can account for more than 90% of the total cancer size (118, 119). Accordingly, MSC- or CAF-targeted therapies may be more effective than CSC-targeted therapy in desmoplastic cancers. Furthermore, CAFs are often localized to the margin of the glands or the cancer cell nests and close to blood vessels, therefore, drug delivery to these cells is easy (120). By contrast, CSCs are located farther away from blood vessels in desmoplastic cancers. In fact, CAFs per se is a major barrier for the delivery of nanoparticles and drugs to tumor cells (140, 141). Indeed, studies have been shown the importance of the spatial distribution of cells in the treatment of desmoplastic cancers (142). Collectively, in the treatment of desmoplastic cancers, targeting the communication between MSCs or CAFs with CSCs is more reasonable, possible, and clinically promising than the direct targeting of CSCs (121).

### 3.4 Specifically Targeting of CSCs by TRAIL-Expressing MSCs

By genetic engineering of MSCs, specific cancer cells can be targeted. For instance, it has been shown that TNF-related apoptosis-inducing ligand (TRAIL)-expressing MSCs specifically target tumor cells in lung carcinoma, therefore reducing chemoresistance, cancer aggressiveness, and relapse (124). TRAIL is a member of the TNF ligand family that can cause apoptosis through the interaction of its death receptors. TRAIL selectively initiates apoptosis of a variety of cancer cells and transformed cells, but not most normal cells, and therefore it has attracted great interest as a promising factor in cancer therapy (143). Several studies have shown the ability of TRAIL-expressing MSCs homing to the tumor site (144–146). TRAIL is a protein that causes apoptosis of tumor cells, without injuring the normal cells, by binding to specific TRAIL receptors and stimulation of the extrinsic apoptosis pathway (147). The activation of the NF-κB signaling pathway (148) and the overexpression of the TRAIL decoy receptors can contribute toward TRAIL resistance in normal cells (149). TRAIL-induced apoptosis has been shown in CD133-positive glioma cells (125). However, Capper et al. showed that CD133-positive neuro-sphere-forming glioma cells were completely resistant to TRAIL (150). It has been reported that TRAIL-expressing MSCs can target both stem-like, side population (SP) cells, and non-SP cells, and in combination with traditional chemotherapies show a synergistic effect in apoptosis induction (151). It has been also shown that physiological levels of TRAIL in MSC-EV was not effective in inducing apoptosis in NSCLC cells (152). High expression of death receptor 4 (DR4) and DR5 were observed in liver and lung cancer-derived CSCs, representing their contribution to CSCs TRAIL sensitivity (153, 154). Activation of both intrinsic and extrinsic apoptosis pathways through extracellular stimulation by TRAIL may induce further effects, especially for chemo-resistant CSCs that show resistance to intrinsic apoptosis pathways (155).

### 3.5 Exosome-Based Cancer Therapy

Numerous miRNAs are differentially expressed in CSCs, which can be used as potential targets in the treatment of cancer (156). It has been recently shown that upregulation of miR-150 and miR-142-3p in BCSCs compared to non-tumorigenic tumor cells can be related to clonogenicity and tumorigenicity of BCSCs (157). Thus, using complementary miR-142 inhibitors in BCSCs could reduce tumor growth. Exosomes derived from MSCs can act as an extracellular messenger to introduce exogenous LNA-antimiR-142-3p to breast cancer stem-like cells to inhibit miR-142-3p and decrease the tumorigenicity, proliferation, and colony formation ability of the cancer stem-like cells (126). MSCs are one of the main sources of exosomes that are especially considered in clinical applications. Indeed, the biological activity of MSCs-derived exosomes is likely akin to the effects mediated by MSCs themselves. Thus, unlike MSCs, the exosomes can be exploited as cell-free carriers, which do not have a risk of tumorigenesis (158). Targeting the CSCs by using the exosomes can introduce a novel tool for destroying CSCs in anti-cancer therapies (126).

### 3.6 CSCs Targeting by Immunotherapy

Recently, immunotherapy has gained great attention in cancer treatment (159). Many studies have used immunotherapeutic approaches to target cancer stem cells. Immune checkpoint inhibitors, antibody-based and adoptive cell therapy approaches are used for CSCs targeting (**Table 2**). CAR-T cell therapy, as an adoptive cell therapy method, is used for CSC-directed immunotherapy by targeting CD20 (NCT03398967), CD123 (NCT02937103), CD19 (NCT03398967) positive cells. Various immune checkpoint blocking agents, such as CTLA-4 inhibitors (Ipilimumab that is approved by the FDA) (1) and PD1/PD-L1 inhibitors: atezolizumab (2), avelumab (3), cemiplimab (4), and nivolumab (5) are undergoing clinical trials. Targeting surface antigens of CSC, such as EpCAM/CD3 (NCT00189345), CD123 (NCT02472145), CD33 (NCT03727750) are other strategies in CSC-directed immunotherapy.

### 3.7 The Clinical Challenges in MSC-Based Therapies

Various factors affect the clinical outcome of MSCs-based therapies. One of the influencing factors is variables related to the preparation of the MSC product. Donor variations such as genetics, age, health status, gender can affect the potency of MSCs (160). In addition, MSCs tissue of origin (161), isolation methods (162), the culture conditions (163), cryopreservation, and thaw/culture rescue protocols (164, 165) causing additional variations in potency of MSCs. The administration of MSCs is another variable that can affect the residence time, viability, and homing of MSCs. This variable includes the following: the inoculation site (dense/non-dense tissue), administration route (local/systemic), injection/infusion buffer, injection device features (needle size/geometry), and cell carrier materials (166, 167). MSC recipients are the third important variable that can affect the therapeutic outcome. Which can refer to the following: host cytotoxic responses against MSCs (168), and the host disease/severity which can lead to highly variable microenvironmental factors such as hypoxia, inflammation status, and ECM that influence the function of MSCs (169).

## 4 Conclusions and Perspective

MSCs can modify the stroma, and helping to establish a tissue microenvironment that favors the restoration of CSCs and tumor progression, as well as crosstalk between cancer cells and MSCs in the microenvironment, promotes a CSC phenotype in cancer cells. Since crosstalk between CSCs and MSCs promote drug resistance, mediates metastasis, and induces tumor growth by inducing CSC phenotype in cancer cells and restoration of CSCs, it is suggested that blockade of the crosstalk of MSCs with CSCs can provide a new avenue to improving the cancer therapeutic tools. Indeed, targeting the communication between MSCs with CSCs is more reasonable, possible, and clinically promising than the direct targeting of CSCs in the treatment of desmoplastic cancers.

## Author Contributions

Conception and design: ZH, JT, and YJ. Collection and assembly of data: YJ, WL, and LZ. Manuscript writing: YJ, WL, LZ, and ZH. Made critical revisions: WL, ZH, and JT. All authors reviewed and approved of the final manuscript.

## Funding

This study was supported by the Public Welfare Application Plan Project of Shaoxing (2018C30109); Project of Shaoxing Medical Key Discipline Construction Plan (2019SZD06); Health and Family Planning Commission of Zhejiang province (2018KY831). The funder had no role in study design, data collection and analysis, decision to publish, or preparation of the manuscript.

## Conflict of Interest

The authors declare that the research was conducted in the absence of any commercial or financial relationships that could be construed as a potential conflict of interest.

## Publisher’s Note

All claims expressed in this article are solely those of the authors and do not necessarily represent those of their affiliated organizations, or those of the publisher, the editors and the reviewers. Any product that may be evaluated in this article, or claim that may be made by its manufacturer, is not guaranteed or endorsed by the publisher.

## Glossary

**Table:**

AR	androgen receptor
BCSCs	breast cancer stem cells
BM-MSCs	bone marrow mesenchymal stromal cells
BMP	bone morphogenetic protein
CAFs	cancer-associated fibroblasts
CA-MSCs	cancer associated MSCs
CCL5	c-c motif chemokine ligand 5
CD	cluster of differentiation
COX-2	cyclooxygenase-2
CRC	colorectal cancer
CSCs	cancer stem cells
CXCL	chemokine (C-X-C motif) ligand
CXCR	CXC chemokine receptors
DNA	deoxyribonucleic acid
DR	death receptor
ECM	extracellular matrix
EGF	epidermal growth factor
EMT	epithelial–mesenchymal transition
ERK	extracellular signal-regulated kinase
EVs	extracellular vesicles
EWS	ewings sarcoma
FAK	focal adhesion kinase
gCSCs	glioma cancer stem cells
GP130	glycoprotein 130
GPR-77	G protein-coupled receptor 77
GRO-α	growth-regulated oncogene-α
HGF	hepatocyte growth factor
HNSCC	head and neck squamous cell carcinoma
IFNγ	interferon γ
IGF	insulin-like growth factor 1
IL	interleukin
IL-6R	interleukin 6 receptor
LDM	low-dose metronomic
LGR-5	leucine-rich repeat-containing G-protein coupled receptor 5
LNA	locked nucleic acid
MCs	mesenchymal cells
miRNAs	microRNAs
MMP	matrix metallopeptidase
mPGES-1	microsomal Prostaglandin-E synthase-1
MPO	myeloperoxidase
MSCs	mesenchymal stem cells
mTOR	mammalian target of rapamycin
NF-κB	nuclear factor kappa B
NO	nitric oxide
NOD/SCID	nonobese diabetic/severe combined immunodeficiency
NSCLC	non-small cell lung carcinoma
OPN	osteopontin
PCa	prostate cancer
PDAC	pancreatic ductal adenocarcinoma
PDGF	platelet-derived growth factor
PGE-2	prostaglandin-E2
SDF-1	stromal cell-derived factor 1
SP	side population
SSM	stem/serrated/mesenchymal
STAT	signal transducers and activators of transcription
TGF-β	transforming growth factor-β
TNF-α	tumor necrosis factor-α
TRAIL	TNF-related apoptosis inducing ligand
WJMSC	Wharton’s jelly of umbilical cord
